# Investigating the Electronic and Molecular Adsorption Properties of Ti/Co-Doped Boron Carbon Nitride

**DOI:** 10.3390/molecules30091873

**Published:** 2025-04-22

**Authors:** Nada M. Alghamdi, Hind M. Al-qahtani, Amal Alkhaldi, Mohamed M. Fadlallah, Ahmed A. Maarouf

**Affiliations:** 1 Department of Physics, College of Science, Imam Abdulrahman Bin Faisal University, Dammam 31441, Saudi Arabia; nmghamdi@iau.edu.sa (N.M.A.); aalkhaldi@iau.edu.sa (A.A.); 2 Department of Physics, College of Science and Humanities, Imam Abdulrahman Bin Faisal University, Jubail 35811, Saudi Arabia; halqahtani@iau.edu.sa; 3 Physics Department, Faculty of Science, Benha University, Benha 13518, Egypt; 4 Department of Physics, Faculty of Basic Sciences, German University in Cairo, New Cairo City 11835, Egypt

**Keywords:** hexagonal boron nitride, electronic properties, doping, molecular adsorption, ab initio calculations

## Abstract

Two-dimensional (2D) hexagonal boron carbon nitride (*h*-B_*x*_C_*y*_N_*z*_) has garnered a lot of interest in the last two decades because of its remarkable physical and chemical characteristics. Because of the carbon atoms, it has a smaller gap than its cousin, boron nitride, and is hence more appropriate for a wider range of applications. In the frame of density functional theory, we discuss the structural, electronic, and magnetic properties of mono Ti-doped and Co-doped BC_6_N (Ti/Co-BC_6_N) at different sites of substitutional doping (Ti/Co) in the BC_6_N monolayer. The mono substitutional doping at the B (Ti_B_/Co_B_), N (Ti_N_/Co_N_), and two different C (C1 (Ti_C1_/Co_C1_), C2 (Ti_C2_/Co_C2_)) sites, are investigated. The position of the Ti/Co dopant is an important parameter that changes the electronic state, magnetic moment, and adsorption activity of the pristine BC_6_N nanosheet. We find that the adsorption of the gases NO, NO_2_, CO_2_, NH_3_, N_2_, and O_2_ is significantly improved on the doped sheet at all doped positions compared to the adsorption on the pristine structure. The Ti/Co-BC_6_N can adsorb NO and NO_2_ better than CO_2_ and NH_3_. Ti_C1_-BC_6_N and Ti_B_-BC_6_N are the best doped sheets for adsorbing NO and NO_2_, respectively. The CO_2_ and the N_2_ molecules are moderately adsorbed at all doped positions as compared to the other adsorbed molecules. Ti-doped sheets can adsorb the CO_2_, NH_3_, and O_2_ better than the corresponding Co-doped sheets. We also study the adsorption of molecular hydrogen on our single-atom Ti/Co-doped systems, as well as on 4-atom Ti and Co clusters embedded in the BC_6_N sheets. We show that the cluster-embedded sheets can adsorb up to four H_2_ molecules. These novel findings are important for many applications of BC_6_N, including spintronics, gas filtration, molecular sensing, and hydrogen storage.

## 1. Introduction

Two-dimensional (2D) nanostructures have attracted much theoretical and experimental attention since the preparation of graphene in 2004 [1]. The low dimensionality of these materials and the diversity of their composition and structural characteristics may have significant potential for technological use. The development of experimental techniques has increased interest in this field since it raises the possibility of creating novel 2D structures with particular chemical or physical characteristics.

Graphene, which has special electrical, mechanical, and optical characteristics that make it suitable for a wide range of technological applications [2], is arguably the most well-known two-dimensional (2D) material. Nevertheless, several drawbacks of graphene, such as the absence of an electronic gap, have prompted scientists to look for other graphene-like structures [3,4]. Hexagonal boron Nitride (h-BN) [5], Germanene [6], Silicene [7], MXenes [8,9], transition metal dichalcogenides (TMDs) [3,10], and Mo(W)Si_2_N_4_ [11,12,13] are samples of 2D structures that have attracted a lot of attention lately due to their several possible applications: gas sensing [14,15,16], field-effect transistors [17], hydrogen storage [18,19], sustainable ammonia production [20], and photocatalysis [21,22,23].

Since carbon, nitrogen, and boron are neighbor atoms in the periodic table, they can form bonds that result in a variety of (*h*-B_*x*_C_*y*_N_*z*_) compounds [24,25]. This has attracted a lot of attention [26] because substituting carbon for boron or nitrogen may lead to a spectrum of interesting properties without significantly distorting the lattice structure. It has been demonstrated that the graphene lattice can be changed from a semimetal to a semiconductor by adding BN grains, with a band gap created at the vanishing Dirac points [27,28,29]. Therefore, one expects that a wide range of physical characteristics might result from varying the relative compositions of the three elements in *h*-B_*x*_C_*y*_N_*z*_.

Various techniques, including the solvothermal approach, chemical vapor coating, and the chemical reaction approach [30], have been used to synthesize several ternary B-C-N structures (such as BCN, BC_2_N, BC_4_N, BC_6_N, and B_2_CN). Because BC_2_N is predicted to be more difficult than c-BN [27,31] and more chemically and thermally inert than diamond, it has additionally attracted interest from researchers. The applications of BCN are many and include the oxidation of pollutants and colorants, the extraction of hydrogen from water, the creation of transparent photovoltaic cells, UV absorption, optoelectronics, and catalysis for a variety of chemical interactions [32,33].

Implanting graphene quantum dots with atoms of boron and nitrogen gives BC_6_N 2D quantum dots [34], which were found to be semiconducting with a band gap of 1.2–1.3 eV [35,36]. BC_6_N has some of the physical and mechanical features of graphene, including high stiffness and high thermal conductivity [24,36], due to its graphene-like structure. Intensive research is being conducted to customize the characteristics of BC_6_N for a variety of uses. By substitutional and adsorption doping of BC_6_N [35,36], magnetic semiconductor behavior may become metallic, half-metallic, or diluted. Moreover, the BC_6_N electrostatic landscape can be changed by defects such as vacancies, making it more reactive to certain gases [35,36].

Due to their electronic activity, metals can be used to enhance the adsorption and catalytic properties of 2D nanomaterials. Among the different doping elements, titanium stands out due to its diverse chemical reactivity [37]. Ti-doped 2D materials open many applications in various fields. For example, in graphene, Ti has shown enhanced catalytic activity for oxygen reduction, a potential candidate for fuel cells and other microelectronic devices [37]. In TMDs, Ti has been found to improve the performance of semiconductor devices and enhance their gas-sensing properties [38].

Co-doping has also generated much interest in materials research. Co-doped 2D MoSe_2_ [39] can create active sites that enhance electrocatalysts for hydrogen evolution reduction reactions (HER). Doping MoS_2_ with Co forms a more stable 1T phase with much better catalytic activity [40]. The charge–discharge efficiency of black phosphorus nanosheets can reach more than 90% after doping with Co, which promotes its potential for electrochemical hydrogen storage [41]. The Co dopant improves the absorption of SWCNT for SOF_2_ and SO_2_, which makes Co-SWCNT a promising gas sensor for them [42]. The Co-MOF-5-synthesized materials demonstrate greater abilities to adsorb H_2_, CO_2_, and CH_4_ under high pressure than their Co-free homologues [43].

Although studies have been conducted on pristine BC_6_N, further research has to be carried out on structures doped with other atoms to improve the physical and chemical properties of the BC_6_N sheet. In this work, we investigate the electronic and molecular adsorption characteristics of Ti-doped and Co-doped BC_6_N in the frame of density functional theory. A 3 × 3 supercell of BC_6_N (consisting of 72 atoms) is used, and the effect of substitutional Ti-doping and Co-doping on electronic and magnetic properties, as well as the adsorption activity of these systems, is investigated. The unit cell of the studied structure has four symmetrically inequivalent sites: two C atoms (C1 and C2), B, and N. We consider the adsorption of four gases (NO, CO_2_, NO_2_, and NH_3_) on the pristine and doped systems.

## 2. Results and Discussion

**Figure 1:**
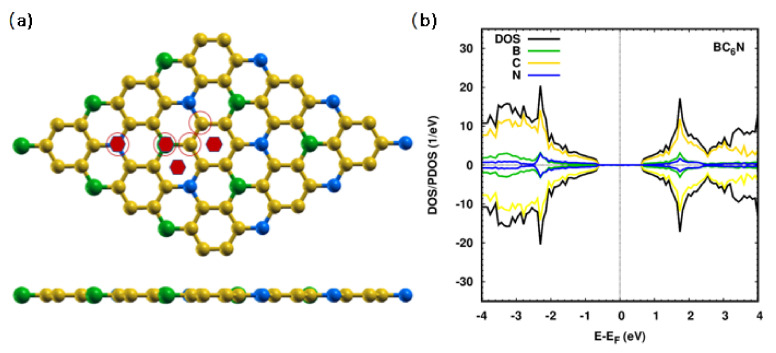
A 3 × 3 optimized BC_6_N pristine monolayer: (**a**) top/side view. We refer to C, B, and N atoms by yellow, green, and blue spheres, respectively. Gas-adsorption positions are indicated with the solid red hexagons. Notation for different sites is as follows: atop N (TN), atop B (TB), atop the BNC hexagonal (H1), and atop the C hexagonal (H2). The red circles refer to the Ti/Co substitutional dopant positions, and (**b**) DOS/PDOS for pristine BC_6_N monolayer.

To study the effect of doping on the structural, electronic, magnetic, and adsorption properties of BC_6_N, we use a 3 × 3 supercell. The optimized atomic structure of a monolayer is shown in Figure 1a with the bond lengths of C-C, C-B, and C-N being 1.41 Å, 1.47 Å, and 1.46 Å, respectively, which agree with previous studies [35,36,44,45]. The four distinct sites of the BC_6_N unit cell are shown: two are at the C1 and C2 sites, and two are at the N and B sites (all marked with red circles).

Figure 1b illustrates the density of states (DOS)/projected DOS (PDOS) of the pristine monolayer. It is semiconducting with a band gap of 1.3 eV, which is in good agreement with published work [35,36,44,45]. The band gap value of BC_6_N indicates that it may be a potential candidate structure for optoelectronic devices. The C 2*p* states are the main contributors to the valence band (VB) and conduction band (CB), with little contribution from the N 2*p* states in the VB, and B 2*p* states in the CB. Charge analysis shows that N and C1 gain some charge, while B and C2 lose some charge.

### 2.1. Ti-Doped and Co-Doped BC_6_N

**Figure 2:**
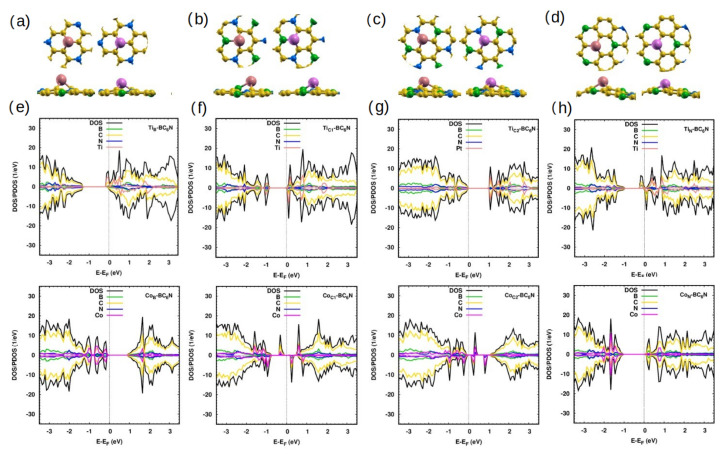
Ti/Co-BC_6_N monolayers with different substitutional doping positions: (**a**–**d**) optimized Ti_B_/Co_B_, Ti_C1_/Co_C1_, Ti_C2_/Co_C2_, and Ti_N_/Co_N_ structures, top/side view. The light-coral and magenta balls indicate the Ti and Co atoms, respectively. (**e**–**h**) The corresponding DOS/PDOS.

The relaxed Ti-doped and Co-doped BC_6_N (Ti/Co-BC_6_N) sheets are shown in Figure 2. Systems with doping at the B ((Ti_B_/Co_B_)), C1 (Ti_C1_//Co_C1_), C2 (Ti_C2_/Co_C2_), and N (Ti_N_/Co_N_) sites exhibit some distortion around the Ti/Co dopant atom, with average nearest doping distances of 1.95 Å/1.76 Å, 1.98 Å/1.8 Å, 1.9 Å/1.8 Å, and 2 Å/1.8 Å respectively. The dopants protrude out of the plane by 1.70 Å/1.45 Å for the first three sites and by 1.8 Å/1.5 Å for the N site (Table 1). The average angles made at the dopants by their nearest neighbors are also shown. The formation energies indicate that Ti_N_/*Co*_N_-BC_6_N has slightly smaller energy compared to other doped nanosheets. All doped systems have thermal stabilities compared to that of the pristine sheet (Table 1). The charge transfer (ΔQ) between the dopant and the sheet of all the considered structures is presented in Table 1.

We now inspect the DOS of the Ti/Co-doped systems (Figure 2e–h). Ti_B_-BC_6_N has an asymmetric DOS. The CB has contributions from the 4*d* Ti states right below the Fermi energy and up to ∼0.6 eV. The C states dominate the VB as in the pristine structure. The structure is metallic as there is no gap in the two spin channels, and the asymmetry of those channels indicates that the sheet is magnetic with 0.9 *μ*_B_. In the case of Ti_C1_- and Ti_C2_-BC_6_N, the structure is non-magnetic, as is obvious from the identical channels of the DOS. The band gaps decrease to 0.9 eV and 1.1 eV, compared to pristine BC_6_N because of the contribution of Ti 4d states, as in the CB, The effect of substitutional doping of Ti_C1_ is more significant in the band gap value than the corresponding effect for Ti_C2_. For Ti_N_-BC_6_N, the structure is a half-metal with no spin-up gap and a spin-down gap of 1.2 eV. The Fermi energy is shifted to the CB. The structure is magnetic with a moment of 0.92 *μ*_B_, as illustrated by the asymmetrical spin-up and spin-down components.

For the DOS of Co_B_- and Co_N_-BC_6_N and as compared to the pristine, many states are created above the VB with a dominated Co 3d states from −1.2 eV to −0.2 eV for Co_B_-doping and from −1.5 eV to −1.2 eV for Co_N_-doping. The contribution of Co-states in the VB is larger than in the CB, which contrasts with the effect in the case of Ti_B_. The DOS of Co_N_-BC_6_N is disturbed more than the Co_B_-BC_6_N. The structures are semiconducting with a band gap of 1.2 eV for both structures. The symmetrical effect of Co-states in both spin directions refers to the zero magnetic moment.

Both Co_C1_- and Co_C2_-BC_6_N monolayers are dilute magnetic semiconductors (DMSCs) due to the significant contributions of Co 3d states with a spin-up (down) band gap of 0.60 eV (1.1 eV) for Co_C1_-doping and 0.4 eV (0.3 eV) for Co_C2_-doping. The spectra of the Co_C_ doped structures are more disturbed than the spectra of systems doped at the B and N positions. The Co 3d contributions are noticed at the top of the VB, in the gap, and at the bottom of the CB. Doping at the C positions changes the magnetic moment to 1 *μ*_B_ due to the asymmetry of the DOS of the spin-up and spin-down components.

Experimental techniques such as UV-Vis absorption spectroscopy can be used to estimate the band gaps, while X-ray photoelectron spectroscopy and ultraviolet photoelectron spectroscopy can provide information on the valence and conduction band positions. In addition, scanning tunneling spectroscopy can offer a direct measurement of the electronic structure. These methods have been widely used in the literature to investigate the band structure of 2D materials [46], including boron–carbon–nitride-based systems.

### 2.2. Adsorption on Ti-/Co-BC_6_N Nanosheets

Now we address the gas-adsorption properties of our structures. We consider six gases NO, NO_2_, CO_2_, NH_3_, O_2_, and N_2_. The initial positions of each gas above the considered sheets are (@_B_), (@_N_), (@_H1_), and (@_H2_) for pristine BC_6_N, and (@_B_, @_C1_, @_C2_, and @_N_) for the doped Ti/Co-BC_6_N sheets. The gas-sheet systems are structurally relaxed. We then compute the adsorption energies and the electronic structures of the investigated nanosheets compared to the adsorption results on pristine structures.

#### 2.2.1. NO Gas Adsorption

The bond length of N-O is 1.16 Å, which agrees well with previous publications [8]. The NO gas is adsorbed very weakly ($E_{ad}<0.2\mathrm{eV}$) above the pristine sheet at all adsorption positions [47] (Table 2). Therefore the NO gas adsorption on the sheet has negligible effects on the DOS of the pristine system.

When we use the Ti/Co-doped structures, the results are completely different. The Ti/Co dopant’s closest distance from the gas’s N atom is 2.0 Å/1.7 Å, 1.9 Å/1.7 Å, 1.9 Å/ 1.7 Å and 2.0 Å/1.8 Å for NO@Ti_B_-/Co_B_-, NO@Ti_C1_-/Co_C1_-, NO@Ti_C2_-/Co_C2_- and NO@Ti_N_-/Co_N_-BC_6_N, respectively. This means that the interaction between the NO gas and the doped sheets is stronger than that of the pristine sheet because the distances are much closer for the doped sheet compared to the pristine structure.

The distance between the N atom and the Ti-BC_6_N is larger than the corresponding distance in the case of Co-BC_6_N. Furthermore, the N-O length increases to 1.2 Å/1.2 Å for NO@Ti_B_/Co_B_, NO@Ti_C1_/Co_C1_ and @Ti_N_/Co_N_, and 1.3 Å/1.2 Å for NO@Ti_C2_/Co_C2_. The charge transfer for the Ti-doped system is higher than the corresponding charge transfer for the Co-doped system at the same position, and with higher adsorption energies: 2.9 eV/ 2.3 eV, 3.4 eV/1.9 eV, 2.4 eV/3.2 eV, and 2.6 eV/2.7 eV for Ti_B_-/Co_B_-, Ti_C1_-/Co_C1_, Ti_C2_-/Co_C2_, and Ti_N_-/Co_N_-BC_6_N sheets. The Ti dopant is more effective in adsorbing the NO at Ti_B_ and Ti_C1_ than the corresponding cases using Co dopant. However, the Co dopant can adsorb the NO at Co_C2_ and Co_N_ more than the corresponding Ti cases.

**Figure 3:**
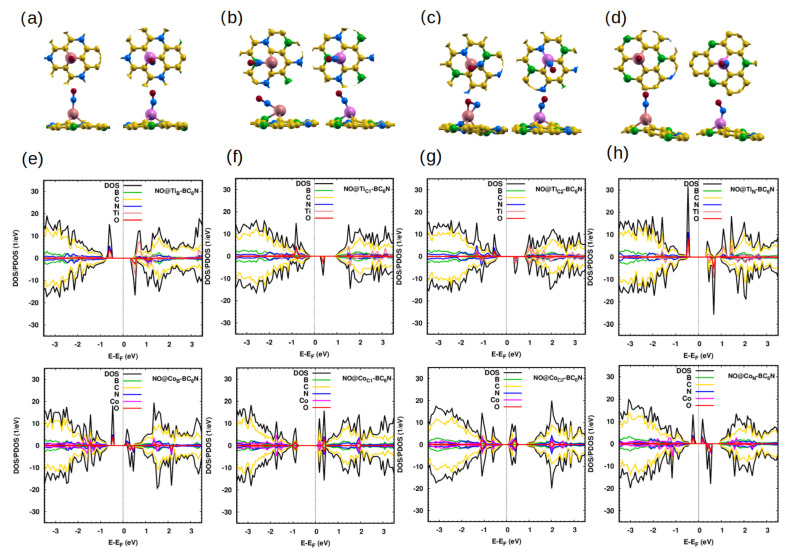
(**a**–**d**) Optimized monolayers of NO@Ti/Co-BC_6_N with different substitutional doping positions, top and side view: (**a**) NO@Ti_B_/Co_B_, (**b**) NO@Ti_C1_/Co_C1_, (**c**) NO@Ti_C2_/Co_C2_, and (**d**) NO@Ti_N_/Co_N_. (**e**–**h**) The corresponding DOS/PDOS of the NO@Ti/Co−BC_6_N monolayers.

Figure 3e–h show the DOS/PDOS of NO@Ti_B_ and NO@Ti_N_-BC_6_N. The top/bottom of the VB/CB is disturbed due to the contribution of the N and O 2p states, such as for the spin-up/spin-down channel for the top/bottom of the VB/CB. The structures are DMSC with a band gap up (down) of 1.0 eV (1.3 eV) for NO@Ti_B_-BC_6_N and 1.0 eV (1.2 eV) for NO@Ti_N_-BC_6_N, and with a magnetic moment of 2 *μ*_B_. The adsorption of NO gas on the Ti_C1_ and Ti_C2_ sites creates states at 0.4 eV and 0.6 eV, respectively. The NO adsorbed gas converts the Ti_C1_-BC_6_N and Ti_C2_-BC_6_N from a SC state to a DMSC state and nonmagnetic to magnetic with 1 *μ*_B_. The spin-up (down) band gaps for Ti_C1_-BC_6_N and Ti_C2_-BC_6_N sheets are 1.2 eV (0.6 eV) and 1.1 eV (0.9 eV).

For adsorbed NO gas on the Co-BC_6_N at different adsorption positions, the contribution of N and O 2p states has a significant effect around the Fermi energy of the Co-BC_6_N sheet. The adsorbed NO molecule converts the SC state of Co_B_- and Co_N_-BC_6_N to a DMSC with spin-up (down) band gaps of 0.5 eV (1.0 eV) and 0.4 eV (0.9 eV), respectively. In addition, the NO molecule converts the nonmagnetic sheet to a magnetic sheet by 1 *μ*_B_ for Co_B_- and Co_N_-BC_6_N. However, the molecule converts the Co_C1_- and Co_C2_-BC_6_N to an SC with a band gap of 1.1 eV and 0.4 eV, respectively. The NO molecule converts the magnetic sheet to a nonmagnetic sheet for Co_B_- and Co_N_-BC_6_N.

#### 2.2.2. NO_2_ Gas Adsorption

We now discuss the adsorption NO_2_ adsorption results. In the NO_2_ isolated molecule, the angle and the bond length are 134° and 1.2 Å, respectively [8]. The adsorption of the molecule is extremely weak (0.1 eV) at all sites of the pristine system [47]. Due to that weak adsorption, its influence on the electronic properties of the pristine sheet is very small [47].

For the adsorption of NO_2_ on the Ti/Co-doped monolayers, in all structures, the bond length of N-O is ∼1.3 Å. We also find that the O-N-O angle decreases to 118.8 °/111.8°, 120.9°/123.7°, 110.8°/108.9°, and 121.1°/110.7° for Ti_B_/Co_B_-, Ti_C1_/Co_C1_-, Ti_C2_/Co_C2_-, and Ti_N_/Co_N_-BC_6_N, respectively. The significant interaction between Ti/Co-doped sheets and adsorbed NO_2_ is reflected in the change in angle. This is further supported by the charge transfer between the sheets and the gas (∼0.5*e*/0.4*e*) and the reduced distance between NO_2_ and the Ti/Co atom (Table 3). Similar to the adsorption of NO gas, the Ti_B_-, Ti_N_- and Ti_C1_-BC_6_N can adsorb the NO_2_ molecule better than the Co_B_-, Co_N_- and Co_C1_-BC_6_N. On the other hand, Co_C2_-BC_6_N can adsorb NO_2_ better than Ti_C2_-BC_6_N.

**Figure 4:**
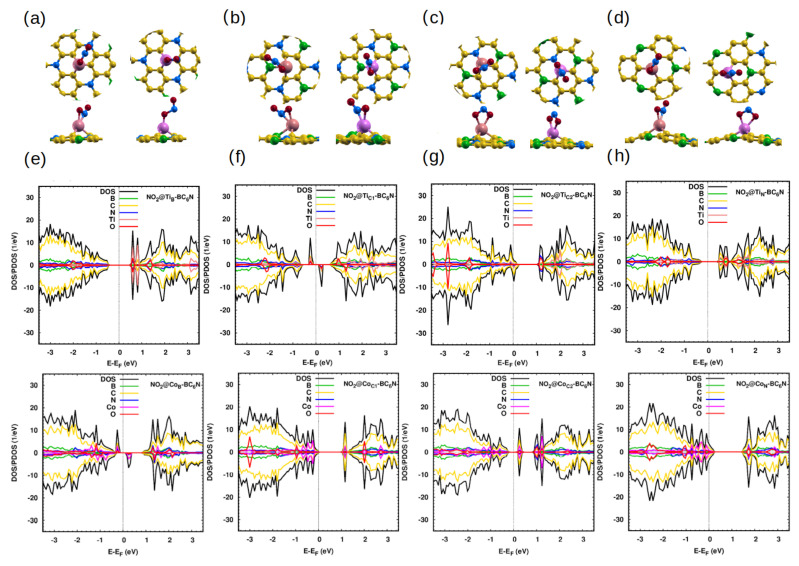
(**a**–**d**) Optimized monolayers of NO_2_@Ti/Co-BC_6_N with different substitutional doping positions, top and side view: (**a**) NO_2_@Ti_B_/Co_B_, (**b**) NO_2_@Ti_C1_/Co_C1_, (**c**) NO_2_@Ti_C2_/Co_C2_, and (**d**) NO_2_@Ti_N_/Co_N_. (**e**–**h**) The corresponding DOS/PDOS of the NO_2_@Ti/Co-BC_6_N structures.

Figure 4e–h show the effect of NO_2_ on the DOS/PDOS of the Ti-BC_6_N sheets. The contribution of the O 2p states is more significant than the N 2p states of the gas. Adsorption changes the magnetic properties and the electronic state of Ti-BC_6_N, from metal to SC for NO_2_@Ti_B_ and from half-metal to SC for NO_2_@Ti_N_ with a band gap of 1 eV for both structures, from SC to DMSC for NO_2_@Ti_C1_ with spin-up/down band gap of 0.6 eV/ 0.9 eV with a magnetic moment of 1 *μ*_B_, and from SC to metal for NO_2_@Ti_C2_. Regarding the DOS/PDOS of NO_2_ on the Co-doped nanosheets (Figure 4), the NO_2_ gas converts the Co_B_- and Co_N_-BC_6_N from SC to DMSC (with spin-up/down band gap of 1.2 eV/0.4 eV for Co_B_-BC_6_N and magnetic moment of 1 *μ*_B_) and metal state, respectively. However, the NO_2_ adsorption changes the state of the Co_C1_- and Co_C2_-BC_6_N systems from DMSC to SC with a gap of 1.2 eV and 0.5 eV, respectively.

#### 2.2.3. CO_2_ Gas Adsorption

The last triatomic gas we examine is CO_2_. Adsorption on the pristine system is extremely weak (0.2 eV) [47]. The story in the Ti/Co-BC_6_N is completely different. The doped monolayer adsorbs the CO_2_ at a distance of ∼2 Å/2.1 Å, and the O-C-O angle decreases significantly. The closest atom of the molecule to the sheet is the O atom except for Co_B_- and Co_C1_-BC_6_N sheets. The charge transfer and the adsorption energy of CO_2_ on Ti-doped sheets are larger than that of Co-doped sheets (Table 4).

**Figure 5:**
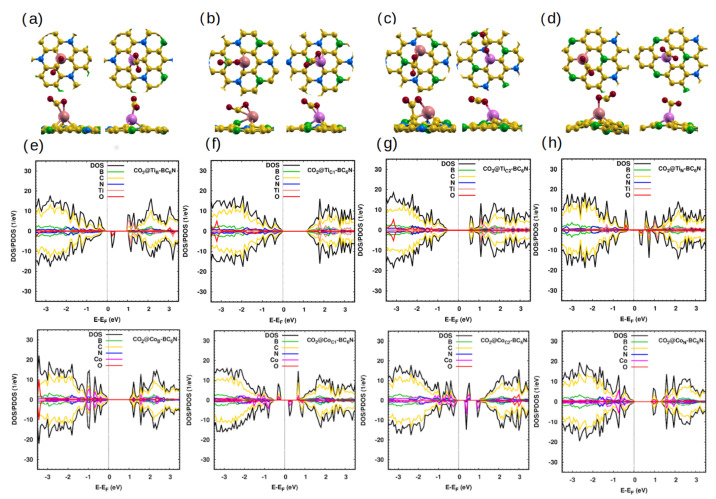
(**a**–**d**) Optimized structures of CO_2_@Ti/Co-BC_6_N at different substitutional doping positions, top and side view: (**a**) CO_2_@Ti_B_/Co_B_, (**b**) CO_2_@Ti_C1_/Co_C1_, (**c**) CO_2_@Ti_C2_/Co_C2_, and (**d**) CO_2_@Ti_N_/Co_N_. (**e**–**h**) The corresponding DOS/PDOS of the CO_2_@Ti/Co-BC_6_N structures.

The PDOS of the CO_2_@Ti_B_- and CO_2_@Ti_N_-BC_6_N systems show that they are DMSCs with spin-up/down and gap of 1.1 eV/0.4 eV and 1.1 eV/0.7 eV, respectively, while CO_2_@Ti_C1_- and CO_2_@Ti_C2_-BC_6_N sheets are SCs with gap of 1.2 eV and 1.1 eV, respectively. On the other hand, CO_2_@Co_B_- and CO_2_@Co_N_-BC_6_N are SCs with the same band gap of 1.1 eV and CO_2_@Co_C1_- and CO_2_@Co_C2_-BC_6_N are DMSCs with spin-up/down band gap of 0.9 eV/0.9 eV and 0.6 eV/0.4 eV, respectively (Figure 5e–h).

#### 2.2.4. NH_3_ Gas Adsorption

**Figure 6:**
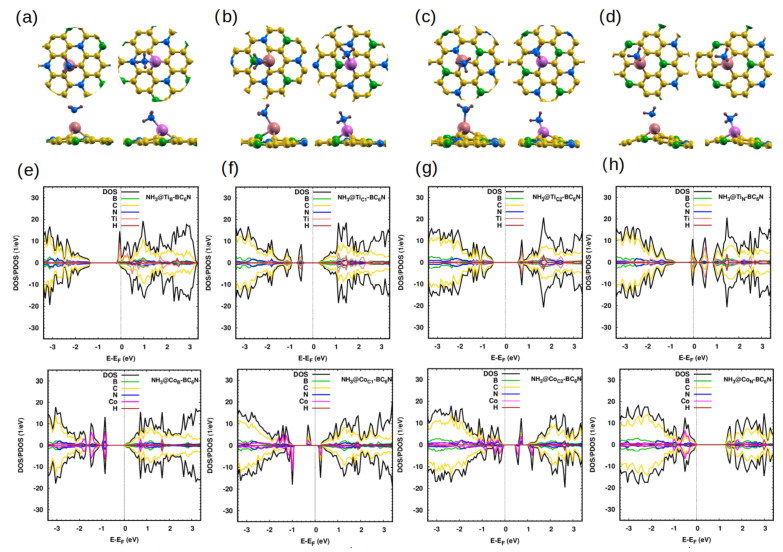
(**a**–**d**) Optimized monolayers of NH_3_@Ti/Co-BC_6_N at different substitutional doping sites, top and side view: (**a**) NH_3_@Ti_B_/Co_B_, (**b**) NH_3_@Ti_C1_/Co_C1_, (**c**) NH_3_@Ti_C2_/Co_C2_, and (**d**) NH_3_@Ti_N_/Co_N_. (**e**–**h**) The corresponding DOS/PDOS of the NH_3_@Ti/Co-BC_6_N structures.

NH_3_ is adsorbed very weakly on the pristine system (0.2 eV) [47]. For the Ti/Co-doped sheets, the closest atom of the sheets is the N atom, and the distance is smaller than the corresponding distance in the pristine sheet (Table 5). As with the previous systems, the Ti/Co-doped system greatly enhances the charge transfer and the adsorption of NH_3_. It is chemisorbed with an average energy of ∼1.4 eV/1.3 eV. The electronic structure becomes metal for NH_3_@Ti_B_- and NH_3_@Ti_N_-BC_6_N, SC with a band gap of 0.8 eV and 1.1 eV in both spin directions for NH_3_@Ti_C1_-BC_6_N and NH_3_@Ti_C2_-BC_6_N, respectively (Figure 6). For Co-doping, NH_3_@Co_B_-, NH_3_@Co_N_-BC_6_N are semiconductors with a band gap of 1.0 eV and 1.3 eV, respectively. NH_3_@Co_C1_-, NH_3_@Co_C2_-BC_6_N are DMSC with spin-up/down band gap of 0.5 eV/1.2 eV and 0.7 eV (0.6 eV), respectively (Figure 6e–h).

#### 2.2.5. N_2_ Gas Adsorption

The fifth molecule that we study is nitrogen (N_2_). The N-N bond length is 1.11. N_2_ gas is adsorbed very weakly by 0.09 eV above the pristine surface at all adsorption positions, which is in agreement with recent studies of N_2_ adsorption on similar systems [48]. As a result, N_2_ gas adsorption on the sheet has a negligible effect on the DOS of a pristine system.

**Figure 7:**
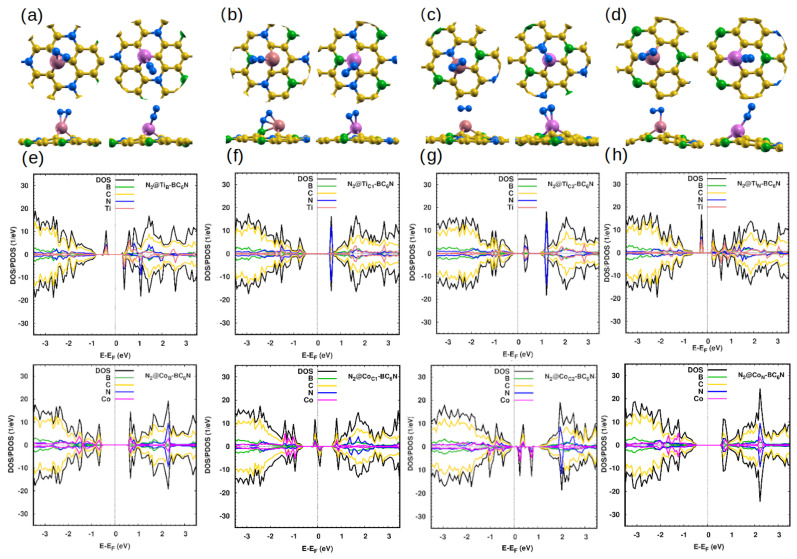
(**a**–**d**) Optimized monolayers of N_2_@Ti/Co-BC_6_N at different substitutional doping sites, top and side view: (**a**) N_2_@Ti_B_/Co_B_, (**b**) N_2_@Ti_C1_/Co_C1_, (**c**) N_2_@Ti_C2_/Co_C2_, and (**d**) N_2_@Ti_N_/Co_N_. (**e**–**h**) The corresponding DOS/PDOS of the N_2_@Ti/Co-BC_6_N structures.

In the doped systems, the distance between the N atom of the gas and the dopant atom ranges from 2.0 Å to 2.9 Å for the Ti systems and from 1.6Å to 2.0 Å for the Co systems (Figure 7a–d). The adsorption energy ranges between ∼0.4 eV and ∼1.3 for Ti-doped systems, and slightly lower for the Co-doped systems (Table 6). The N_2_ adsorption causes some changes in the DOS/PDOS of Ti/Co-BC_6_N sheets (Figure 7a–d). The adsorption changes the metal Ti_B_-BC_6_N and half-metal Ti_N_-BC_6_N into DMSCs with spin-up (down) gaps of 0.6 (1.1) and 0.4 (0.8), respectively. However, the corresponding Co-doped structures remain SC. At the C1 and C2 positions, the Ti-doped structures are SC after adsorption, although with a smaller band gap. The N_2_@Co_C1_-BC_6_N flips from a DMSC to a metal, while the N_2_@Co_C2_-BC_6_N system remains a DMSC.

#### 2.2.6. O_2_ Gas Adsorption

**Figure 8:**
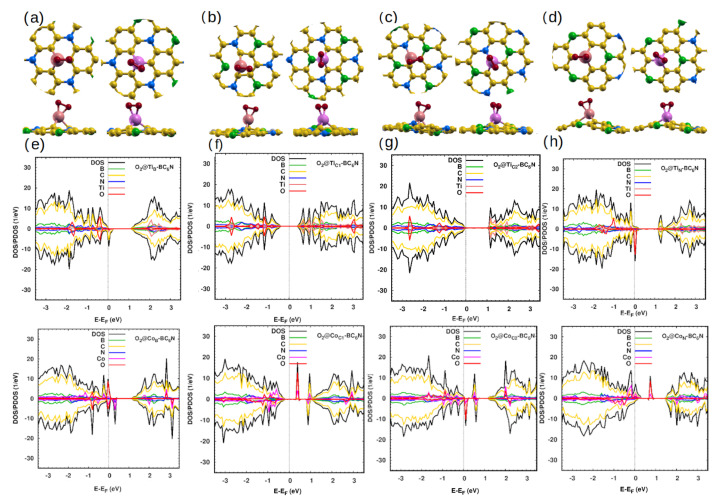
(**a**–**d**) Optimized monolayers of O_2_@Ti/Co-BC_6_N at different substitutional doping sites, top and side view: (**a**) O_2_@Ti_B_/Co_B_, (**b**) O_2_@Ti_C1_/Co_C1_, (**c**) O_2_@Ti_C2_/Co_C2_, and (**d**) O_2_@Ti_N_/Co_N_. (**e**–**h**) The adsorption corresponding to DOS/PDOS of the O_2_@Ti/Co-BC_6_N structures.

The last molecule that we study is O_2_. Oxygen has a bond length of 1.23 Å. Our calculations show that the O_2_ adsorption on the pristine BC_6_N is weak (∼0.1 eV), consistent with previous studies [49]. The distance between the O atom and the dopant atom ranges from 1.78 Å to 1.85 Å for the Ti systems, and from 1.81 Å to 1.94 Å for the Co systems (Figure 8a–d), indicating a stronger interaction than that of the sister molecule N_2_. This is also reflected in the energies, which indicate that O_2_ is chemisorbed to the dopants (Table 7).

Figure 8e–h show the influence of O_2_ on the DOS/PDOS of Ti/Co-BC_6_N systems. Adsorption of O_2_ on Ti_B_-/Co_B_-BC_6_N and Ti_N_-/Co_N_-BC_6_N shifted Fermi energy to the VB, resulting in an asymmetry between spin-up/down spectra with a greater contribution of the O_2_ 2p states in the VB, with the exception of @Co_N_, which has the O_2_ 2p states mainly located in the spin-up gap region. O_2_ adsorption converts the Ti_B_ structure from metal to half-metal, and vice versa for Ti_N_. On the other hand, the structure was changed from SC to metal in both O_2_@Co_B_ and Co_N_. The adsorption of O_2_@Ti_C1_ and Ti_C2_ leaves the two spectra unchanged (spin-symmetric and SC), with band gaps of 0.9 eV and 1 eV. As for the Co systems, O_2_@Co_C1_-BC_6_N remains SC, unlike O_2_@Co_C2_-BC_6_N which changes from SC to metal.

#### 2.2.7. H_2_ Gas Adsorption

**Figure 9:**
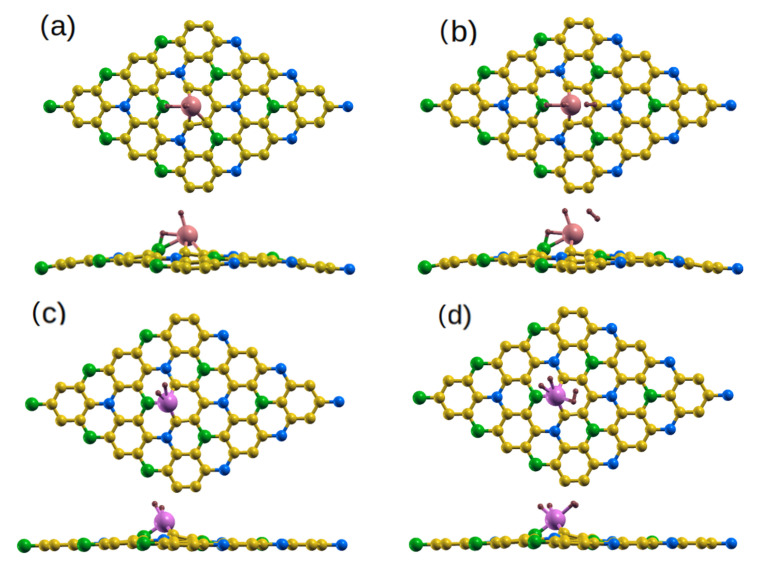
(**a**–**d**) Optimized structures of H_2_@Ti/Co-BC_6_N, with 1 and 2 adsorbed H_2_ molecules, top and side view.

We now explore the potential of molecular hydrogen adsorption on our doped BC_6_N systems. We place the one and two H_2_ molecules consecutively close to the Ti/Co site at the 4 different doping locations. Figure 9 shows two of our systems, Ti_C1_/Co_C1_, with 1 and 2 adsorbed H_2_ molecules. As we see in Table 8, the adsorption strength is intermediate, except for Ti/Co systems at the C1 site. We also notice that the Co-doped systems are slightly better for hydrogen storage than the Ti-doped systems. The relatively small adsorption energy may be attributed to the large saturation of the dopants with the neighboring lattice sites, which leaves little room for the charge transfer necessary for a strong H_2_ adsorption.

**Figure 10:**
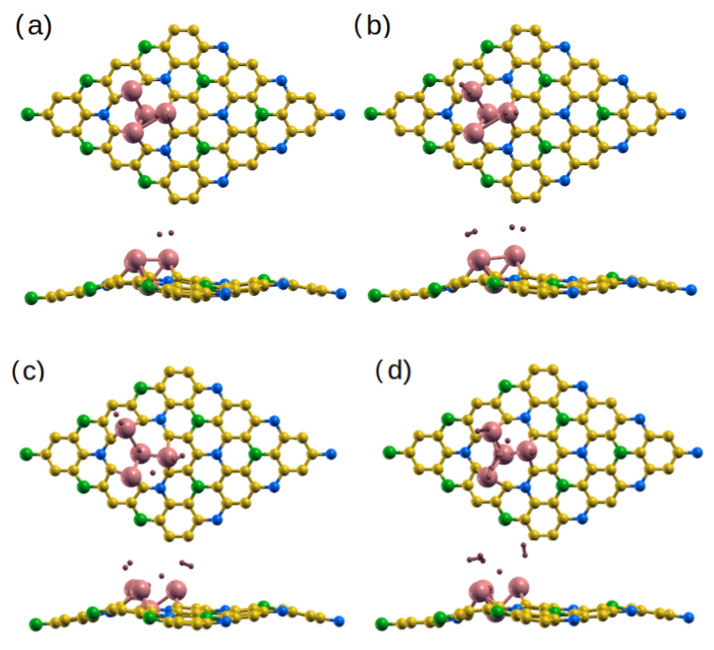
(**a**–**d**) Optimized structures of H_2_@Ti-BC_6_N, with 1, 2, 3, and 4 adsorbed H_2_ molecules, top and side view.

Now that we see that the single-atom doped systems may not be ideal for hydrogen storage, we explore the potential of other Ti- and Co-doped BC_6_N systems. Figure 10 shows our Ti-doped systems, where 4 Ti atoms are implanted in the BC_6_N skeleton, which may be experimentally realized in an implantation scheme. Given the symmetry of our BC_6_N pristine structure, there are two inequivalent locations where the 4-atom cluster can land: one centered around the B site, and the other around the C2 site (Figure 10 shows the systems with the cluster centered around the former). Centering the clusters around the N and C1 sites yields the same systems. We systematically add up to four H_2_ molecules, each time structurally relaxing our systems and obtaining the average H_2_ adsorption energies. The average is taken over the number of H_2_ molecules, according to:

(1) $$E_{\mathrm{ad}}=\frac{E_{nH2+4\mathrm{Ti}/\mathrm{Co}@\mathrm{BC}6N}-E_{4\mathrm{Ti}/\mathrm{Co}@\mathrm{BC}6N}-nE_{H2}}{n},$$

where $E_{\mathrm{Ad}}$ is the average adsorption energy per H_2_ molecule for *n* adsorbed molecules, $E_{nH2+4\mathrm{Ti}/\mathrm{Co}@\mathrm{BC}6N}$ is the total energy of the 4Ti-BC_6_N sheet with *n* H_2_ molecules adsorbed, $E_{4\mathrm{Ti}/\mathrm{Co}@\mathrm{BC}6N}$ is the total energy of the 4Ti/Co-BC_6_N sheet, and $E_{H2}$ is the total energy of a single H_2_ molecule. The H_2_ molecules can initially be placed in many locations around the cluster. The variability in the location may result in adsorption energy of the $n^{\mathrm{th}}$ H_2_ that is higher than that of the preceding molecule. This may also be caused by a dynamic reorientation of the cluster or sheet atoms, resulting in higher adsorption energy. The relaxation process partially addresses the variability in the location by determining the atomic positions of a (possibly local) energy minimum. Therefore, the average adsorption energy per H_2_ molecule is preferred over the adsorption energy of a specific $n^{\mathrm{th}}$ H_2_ addition, as the average accounts for the variability in the location of the hydrogen molecule around the cluster as well as any reorientation of the cluster atoms as more molecules are added.

As we see in Figure 10, the 4-atom Ti cluster protrudes out of the BC_6_N plane, with the BC_6_N sheet becoming slightly non-planar, with a vertical spread of about 1.53 Å. The sheet does not significantly change its shape as H_2_ is added. The average distances of the H_2_ molecules from the closest Ti atom are 1.61 Å, 2.07 Å, 2.07 Å, and 2.4 Å. The average adsorption energies are 0.48 eV, 0.36 eV, 0.85 eV, and 0.72 eV. For the systems where the Ti cluster is centered around the C2 site, the average H-Ti distances are slightly lower, whereas the average energies are higher (Table 9).

**Figure 11:**
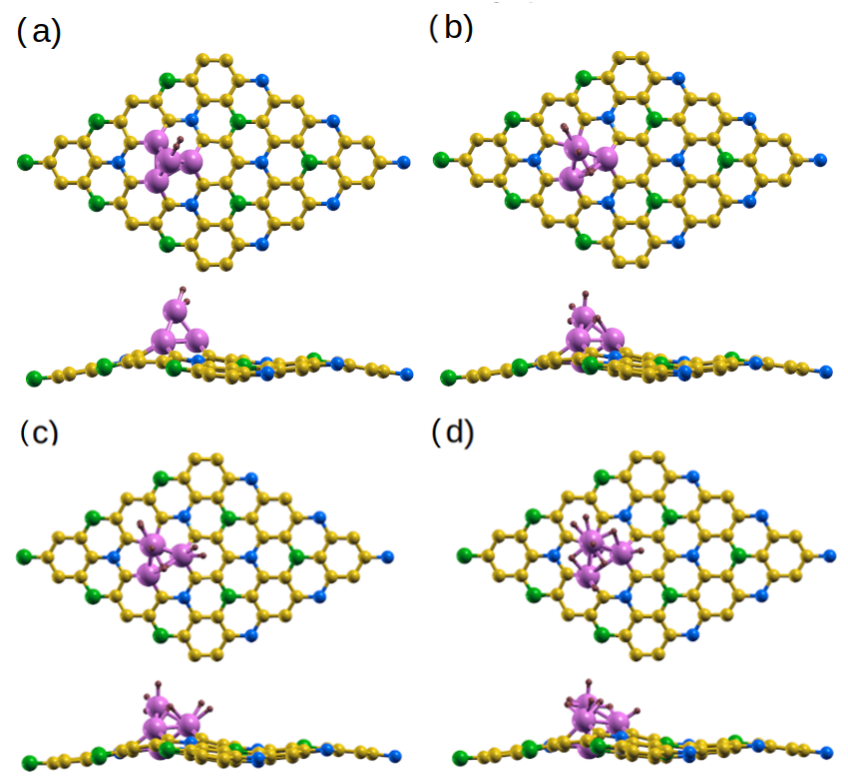
(**a**–**d**) Optimized structures of H_2_@Co-BC_6_N, with 1, 2, 3, and 4 adsorbed H_2_ molecules, top and side view.

The corresponding Co-doped systems are shown in Figure 11. One striking difference from the Ti case is that the Co cluster takes a pyramidal shape, with one Co atom at the top. The sheet suffers some deformation, with a vertical spread of 1.32 Å. The average distances of the H_2_ molecules from the closest Co atom are 1.61 Å, 1.57 Å, 1.59 Å, and 1.54 Å. The average adsorption energies are 0.22 eV, 0.98 eV, 0.73 eV, and 0.60 eV. For the systems where the Co cluster is centered around the C2 site, the average H-Co distance and the average adsorption energies are highly similar (Table 9).

Our results of storing hydrogen on Ti- and Co-doped BC_6_N compares with previous work, which has shown that Ti-decorated *h*-BN monolayers can adsorb up to 5 H_2_ molecules per Ti atom, with an adsorption energy range from 0.68 eV for one molecule and up to 0.22 eV for five molecules [50]. Ti-decorated carbon-doped *h*-BN can store hydrogen at room temperature and mild pressure, with an average adsorption energy of 0.58 eV per molecule [51]. Li-decorated BC_6_N significantly enhances hydrogen storage capacity compared to pristine structures, as Double-sided Li-decorated BC_6_N can adsorb up to eight hydrogen molecules, with adsorption energies of 0.23–0.29 eV [24]. Further research demonstrated that 8Li-decorated BC_6_N could adsorb up to 32 H_2_ molecules [52].

One can determine the Ti/Co-doped BC_6_N’s potential for gas adsorption/filtration by calculating the average adsorption energy of each gas on the various adsorption positions. Table 10 illustrates that NH_3_ and CO_2_ are physisorbed on the Ti/Co-doped systems, whereas NO_2_ and NO are chemisorbed. This shows that our Ti/Co-doped BC_6_N-based sensors would work well for filtering NO_2_ and NO, and to a lesser degree for NH_3_ and CO_2_. Additionally, we have observed that the adsorption of NO_2_, NO, NH_3_, and CO_2_ alters our Ti/Co-doped systems’ band gaps in certain ways that could be used to create sensors for those gases. Consequently, the Ti/Co-BC_6_N systems investigated in this work represent promising candidate materials for NO_2_, No, NH_3_, and CO_2_ filtration and sensing. In addition, the Ti/Co cluster-doped BC_6_N systems show great potential for the adsorption of up to 4 H_2_ molecules.

The Ti- and Co-doped BC_6_N system exhibits promising adsorption characteristics for various gases, suggesting its applicability in gas-sensing, capture, and catalytic processes. The high adsorption energies observed for NO (2.0–3.4 eV) and NO_2_ (2.0–3.4 eV) indicate strong binding, making the material suitable for gas sensing and environmental monitoring of these toxic pollutants. Such sensors could be used to detect nitrogen oxides in industrial emissions and urban environments, improving air quality-control efforts [53]. The moderate adsorption energies for CO_2_ (0.4–2.2 eV) suggest potential applications in carbon-capture and storage (CCS) technologies, where reversible adsorption is crucial for efficient gas separation and sequestration [54].

Additionally, the material’s affinity for NH_3_ (1.2–1.5 eV) and N_2_ (0.5–1.5 eV) suggests it could serve as a selective gas filter or membrane for ammonia removal in industrial processes, such as fertilizer production and waste treatment [55]. The exceptionally high adsorption energy for O_2_ (3.0–5.0 eV) indicates potential applications in oxygen-storage and -release systems, which are critical for medical and industrial applications [56]. These results highlight the versatility of BC_6_N-based materials for next-generation adsorption technologies, combining tunable gas interactions with structural stability.

Experimental studies show that B*_x_*C*_y_*N*_z_* materials exhibit significantly higher gas uptake than graphene, with CO_2_ and CH_4_ adsorption increasing exponentially with surface area, unlike the linear trend in graphene. These findings, supported by theoretical calculations, highlight the superior adsorption capabilities of B*_x_*C*_y_*N*_z_* [57]. Similarly, another study demonstrates that vertically aligned MoS_2_ exhibits enhanced gas adsorption at its edge sites due to a high density of exposed edges, leading to stronger NO_2_ binding [58].

To the best of our knowledge, no experimental measurements have been reported for gas adsorption on Ti- and Co-doped boron carbon nitride systems. However, studies on similar Ti-doped 2D materials may provide useful insights. It has been found that NO gas molecules adsorb on Ti-doped graphene with an energiy of 1.72 eV [59]. Additionally, Ti-doped 2D materials exhibit strong adsorption capabilities for HCHO, CO, and SO_2_ [59], and acetone [60], with adsorption energies reaching up to 0.8 eV. These findings suggest that Ti- and Co-doped boron carbon nitride could also demonstrate promising gas-adsorption properties, which warrants experimental validation.

## 3. Computational Methods

We utilize the spin-polarized first-principles calculations using the Quantum Espresso software (V6.5) [61]. The generalized gradient approximation in the framework of the Perdew–Burke–Ernzerhof functional [62] is used to describe the exchange–correlation interaction. We use a 50 Ry energy cut-off. Our pristine system is a 3 × 3 supercell of BC_6_N monolayer with 72 atoms. The doped monolayers are created by substituting a dopant atom for C (2 positions), B, and N. We take a 20 Å vacuum spacing along the *z*-axis to suppress interactions between neighboring images. A minimum force of 0.001 Ry/Bohr is used to obtain the optimized atomic positions and supercell volume of the considered structures. Van der Waals correction [63,64] is considered in our calculations. Our choice of the *k*-point grid is determined by performing convergence tests on the total energy and electronic structure. A Monkhorst–Pack grid of 12 × 12 × 1 is used for the density of states calculations. Charge transfer from/to the BC_6_N sheets is calculated by the Löwdin method, and we complement this by showing the charge density difference maps (Supplementary Materials). In order to investigate the structural stability of different Ti-BC_6_N and Co-BC_6_N systems, we compute the formation energy per atom, $E_{f}$, as:

(2) $$E_{f}=\frac{E\left(\mathrm{Ti}/\mathrm{Co}\text{-}{\mathrm{BC}}_{6}N\right)+E\left(Y\right)-E\left({\mathrm{BC}}_{6}N\right)-E\left(\mathrm{Ti}/\mathrm{Co}\right)}{n},$$

where E(Ti/Co), $E({\mathrm{BC}}_{6}N)$, E(Y), and $E(\mathrm{Ti}/\mathrm{CO}\text{-}{\mathrm{BC}}_{6}N)$ are the total energies of the isolated Ti/Co atom, the pristine sheet, the isolated removed Y atom (Y = C1, C2, B, or N), and the doped sheet, respectively, and *n* is the number of atoms in the supercell. The adsorption energy of a molecule is calculated by

(3) $$E_{ad}=E\left(\mathrm{sheet}\right)+E\left(\mathrm{molecule}\right)-E\left(\mathrm{sheet}+\mathrm{molecule}\right).$$

where E(sheet+molecule), E(sheet), and E(molecule) are the energies of the sheet with the adsorbed molecule, the sheet without the adsorbed molecule, and the isolated molecule, respectively.

## 4. Conclusions

This study employs first-principles calculations to investigate the structural, electronic, and molecular adsorption properties of Ti/Co-doped BC_6_N monolayers, focusing on various substitutional doping sites (B, N, and two distinct C positions). The stability of these doped structures is assessed via formation energy calculations. The findings indicate that Ti/Co doping significantly alters the electronic states and band gaps of BC_6_N nanosheets. The adsorption behaviors of NO, NO_2_, CO_2_, NH_3_, N_2_, and O_2_ molecules on both pristine and doped BC_6_N structures are analyzed. Doping improves the adsorption activity, particularly for NO, NO_2_, and O_2_ while CO_2_ exhibits weaker adsorption across all doped configurations. Adsorption also influences the electronic and magnetic properties of the doped sheets, potentially transitioning them between metallic, semiconducting, and diluted magnetic semiconducting states, or modifying their band gaps. Furthermore, the study explores H_2_ adsorption on Ti/Co-doped BC_6_N and on structures embedded with 4-atom Ti and Co clusters. Although single-atom doping results in relatively weak H_2_ adsorption, cluster-doped configurations can strongly adsorb up to four H_2_ molecules. These results suggest that Ti/Co-doped BC_6_N monolayers hold promise for applications in gas filtration, hydrogen storage, molecular sensing, and spintronics.

## Figures and Tables

**Table 1:** Ti/Co-BC_6_N: The charge transfer (ΔQ (*e*)), formation energy ($E_{f}$ (meV)), magnetic moment (Mag ($\mu_{B}$)), band gap ($E_{g}$ up/down (dn) (eV)), average distance between the dopant and its nearest neighbors, $\bar{r}$, and average angle between the dopant and its nearest neighbors, $\bar{\theta }$.

Systems	ΔQ	$E_{f}$	Mag	$E_{g}$ (up)	$E_{g}$ (dn)	$\bar{r}$	$\bar{\theta }$
BC_6_N	-	-	0.0	1.3	1.3	-	-
Ti_B_/Co_B_-BC_6_N	0.78/0.13	77/52	0.9/0.0	-/1.2	-/1.2	1.95/1.76	82.92/93.79
Ti_C1_/Co_C1_-BC_6_N	0.82/0.03	88/72	−0.1/1	0.9/0.6	0.9/1.1	1.98/1.8	80.19/91.09
Ti_C2_/Co_C2_-BC_6_N	1.03/0.18	58/65	0.0/1	1.1/0.4	1.1/0.3	1.9/1.79	94.42/91.08
Ti_N_/Co_N_-BC_6_N	0.98/0.17	46/21	0.9/0.0	-/1.2	1.2/1.2	2/1.8	91.32/102.38

**Table 2:** NO@pristine [47] and Ti/Co-BC_6_N monolayers: The closest distance (Å), charge transfer (ΔQ (*e*)), nearest atom (X), adsorption energy ($E_{ad}$ (eV)), magnetic moment (Mag (*μ_B_*)), band gap ($E_{g}$ up/down (dn) (eV)).

Systems	d	X	ΔQ	$E_{\mathrm{ad}}$	Mag	$E_{g}$ (up)	$E_{g}$ (dn)
@_B_BC_6_N	2.8	N	0.0	0.2	1.0	0.4	1.1
@_N_BC_6_N	3.1	N	0.0	0.1	1.0	1.1	0.4
@_H1_BC_6_N	3.1	N	0.0	0.1	1.0	0.3	1.2
@_H2_BC_6_N	2.9	N	−0.1	0.2	1.0	0.5	1.3
@Ti_B_/Co_B_-BC_6_N	2.0/1.7	N/N	−0.4/−0.1	2.9/2.3	2/1	1.0/0.5	1.3/1.0
@Ti_C1_/Co_C1_-BC_6_N	1.9/1.7	N/N	−0.3/−0.1	3.4/1.9	1/0	1.2/1.1	0.6/1.1
@Ti_C2_/Co_C2_-BC_6_N	1.9/1.7	N/N	−0.6/−0.1	2.4/3.2	1/0	1.1/0.4	0.9/0.4
@Ti_N_/Co_N_-BC_6_N	2.0/1.8	N/N	−0.3/−0.1	2.6/2.7	2/1	1.0/0.4	1.2/0.9

**Table 3:** NO_2_@pristine [47] and Ti/Co-BC_6_N: The closest distance (Å), O-N-O angle (*θ*°), charge transfer (ΔQ (*e*)), nearest atom (X), adsorption energy ($E_{ad}$ (eV)), magnetic moment (Mag ($\mu_{B}$)), and band gap ($E_{g}$ up/down (dn) (eV)).

Systems	d	*θ*°	X	ΔQ	$E_{\mathrm{ad}}$	Mag	$E_{g}$ (up)	$E_{g}$ (dn)
@_B_BC_6_N	2.9	126.7	O	−0.1	0.1	0.9	1.4	-
@_N_BC_6_N	3.0	126.9	O	−0.2	0.1	0.9	1.4	-
@_H1_BC_6_N	3.0	126.9	O	−0.1	0.1	−0.9	-	1.3
@_H2_BC_6_N	2.8	128.6	N	−0.1	0.1	0.9	0.3	1.3
@Ti_B_/Co_B_-BC_6_N	2.0/1.8	118.8/111.8	O/O	−0.5/−0.4	3.8/2.1	0/1	1.0/1.2	1.0/0.4
@Ti_C1_/Co_C1_-BC_6_N	1.9/1.8	120.9/123.7	O/N	−0.5/−0.3	3.7/2.0	1/0	0.6/1.2	0.9/1.2
@Ti_C2_/Co_C2_-BC_6_N	2.1/2.0	110.8/108.9	O/O	−0.5/−0.3	2.9/3.4	0.3/0	-/0.5	-/0.5
@Ti_N_/Co_N_-BC_6_N	2.1/2.0	121.1/110.7	O/O	−0.5/−0.4	3.6/2.8	0/0	1.0/-	1.0/-

**Table 4:** CO_2_@pristine [47] and Ti/Co-BC_6_N structures: The closest distance (Å), O-C-O angle (*θ*°), charge transfer (ΔQ (*e*)), nearest atom (X), adsorption energy ($E_{ad}$ (eV)), magnetic moment (Mag (*μ_B_*)), and band gap ($E_{g}$ up/down (dn) (eV)).

Systems	d	*θ*°	X	ΔQ	$E_{\mathrm{ad}}$	Mag	$E_{g}$ (up)	$E_{g}$ (dn)
@_B_BC_6_N	3.3	179.3	C	−0.02	0.2	0.0	1.3	1.3
@_N_BC_6_N	3.2	179.8	C	−0.01	0.2	0.0	1.3	1.3
@_H1_BC_6_N	3.2	179.3	C	−0.02	0.2	0.0	1.3	1.3
@_H2_BC_6_N	3.2	179.3	C	−0.02	0.2	0.0	1.3	1.3
@Ti_B_/Co_B_-BC_6_N	2.0/2.0	139/151.9	O/C	−0.5/−0.3	1.1/0.4	1/0	1.1/1.1	0.4/1.1
@Ti_C1_/Co_C1_-BC_6_N	1.9/1.9	128.1/145.4	O/C	−0.5/−0.3	2.2/0.4	0/1	1.2/0.9	1.2/0.9
@Ti_C2_/Co_C2_-BC_6_N	1.9/2.2	126.6/177.9	O/O	−0.5/0.1	1.2/0.8	0/1	1.1/0.6	1.1/0.4
@Ti_N_/Co_N_-BC_6_N	2.1/2.2	144.7/157.2	O/O	−0.4/−0.2	0.7/0.4	1/0	1.1/1.1	0.7/1.1

**Table 5:** NH_3_@pristine and Ti/Co-BC_6_N nanosheets: The closest distance (Å), H-N-H angle (*θ*°), charge transfer (ΔQ (*e*)), nearest atom (X), adsorption energy ($E_{ad}$ (eV)), magnetic moment (Mag (*μ_B_*)), and band gap ($E_{g}$ up/down (dn) (eV)).

Systems	d	*θ*°	X	ΔQ	$E_{\mathrm{ad}}$	Mag	$E_{g}$ (up)	$E_{g}$ (dn)
@_B_BC_6_N	2.7	102.2	H	−0.01	0.2	0.0	1.3	1.3
@_N_BC_6_N	2.7	106.4	H	−0.01	0.2	0.0	1.3	1.3
@_H1_BC_6_N	2.7	106.2	H	−0.01	0.2	0.0	1.3	1.3
@_H2_BC_6_N	2.8	106.2	H	−0.01	0.2	0.0	1.3	1.3
@Ti_B_/Co_B_-BC_6_N	2.3/2.1	106.6/107.4	N/N	0.1/0.2	1.3/1.2	−0.73/0	-/1	-/1
@Ti_C1_/Co_C1_-BC_6_N	2.2/2	108.5/107.6	N/N	0.2/0.2	1.6/1.2	0/1	0.8/0.5	0.8/1.2
@Ti_C2_/Co_C2_-BC_6_N	2.3/2	107.0/108.2	N/N	0.2/0.2	1.5/1.4	0/1	1.1/0.7	1.1/0.6
@Ti_N_/Co_N_-BC_6_N	2.3/2.1	107.4/107.4	N/N	0.2/0.2	1.3/1.4	0/0	-/1.3	-/1.3

**Table 6:** N_2_@Ti/Co-BC_6_N monolayers: The closest distance (Å), charge transfer (ΔQ (*e*)), nearest atom (X), adsorption energy ($E_{ad}$ (eV)), magnetic moment (Mag (*μ_B_*)), band gap ($E_{g}$ up/down (dn) (eV)).

Systems	d	X	ΔQ	$E_{\mathrm{ad}}$	Mag	$E_{g}$ (up)	$E_{g}$ (dn)
@_TB_BC_6_N	3.29	N	0.01	0.09	0.0	1.2	1.2
@Ti_B_/Co_B_-BC_6_N	2.4/1.84	N/N	−0.34/−0.17	0.94/0.86	1/0	0.6/1.1	1.1/1.1
@Ti_C1_/Co_C1_-BC_6_N	2.04/1.69	N/N	−0.34/−0.27	0.44/0.49	0/0	0.9/-	0.9/-
@Ti_C2_/Co_C2_-BC_6_N	2.89/1.97	N/N	−0.17/−0.27	1.29/0.51	0/1.08	0.4/0.4	0.4/0.2
@Ti_N_/Co_N_-BC_6_N	2.23/1.87	N/N	−0.29/−0.11	0.39/0.66	1/0	0.4/1.1	0.8/1.1

**Table 7:** O_2_@Ti/Co-BC_6_N monolayers: The closest distance (Å), charge transfer (ΔQ (*e*)), nearest atom (X), adsorption energy ($E_{ad}$ (eV)), magnetic moment (Mag (*μ_B_*)), band gap ($E_{g}$ up/down (dn) (eV)).

Systems	d	X	ΔQ	$E_{\mathrm{ad}}$	Mag	$E_{g}$ (up)	$E_{g}$ (dn)
@_TB_BC_6_N	2.8	O	0.17	0.114	0.0	-	-
@Ti_B_/Co_B_-BC_6_N	1.85/1.87	O/O	−0.72/−0.65	4.43/2.36	0.9/1.04	1.1/-	-/-
@Ti_C1_/Co_C1_-BC_6_N	1.82/1.85	O/O	−0.67/−0.58	5.06/3.66	0/−1.02	0.9/0.5	0.9/1
@Ti_C2_/Co_C2_-BC_6_N	1.83/1.81	O/O	−0.63/−0.54	4.63/3.14	0/0.87	1/-	1/-
@Ti_N_/Co_N_-BC_6_N	1.78/1.94	O/O	−0.54/−0.55	3.81/1.96	0.7/−1.7	-/-	-/-

**Table 8:** Average adsorption energies and number (storage) of hydrogen molecules on the Ti/Co-doped sheets.

System	$E_{ad}^{ave}$ Ti/Co (eV)
1H_2_ @Ti_B_/Co_B_-BC_6_N	0.15/0.39
2H_2_ @Ti_B_/Co_B_-BC_6_N	0.2/0.40
1H_2_ @Ti_C1_/Co_C1_-BC_6_N	1.21/0.58
2H_2_ @Ti_C1_/Co_C1_-BC_6_N	0.79/0.39
1H_2_ @Ti_C2_/Co_C2_-BC_6_N	0.002/0.42
2H_2_ @Ti_C2_/Co_C2_-BC_6_N	0.1/0.24
1H_2_ @Ti_N_/Co_N_-BC_6_N	0.05/0.27
2H_2_ @Ti_N_/Co_N_-BC_6_N	0.16/−0.58

**Table 9:** Average adsorption energies of hydrogen molecules on the Ti/Co 4-atom cluster-doped systems.

System	$E_{ad}^{ave}$ Ti/Co-BC_6_N (eV)
1H_2_ @4Ti_B_/4Co_B_-BC_6_N	0.48/0.22
2H_2_ @4Ti_B_/4Co_B_-BC_6_N	0.36/0.98
3H_2_ @4Ti_B_/4Co_B_-BC_6_N	0.85/0.74
4H_2_ @4Ti_B_/4Co_B_-BC_6_N	0.72/0.60
1H_2_ @4Ti_C2_/4Co_C2_-BC_6_N	2.47/0.57
2H_2_ @4Ti_C2_/4Co_C2_-BC_6_N	1.33/1.18
3H_2_ @4Ti_C2_/4Co_C2_-BC_6_N	1.27/0.94
4H_2_ @4Ti_C2_/4Co_C2_-BC_6_N	1.03/0.79

**Table 10:** Average adsorption energies ($E_{ad}^{ave}$ (eV)) on the Ti/Co-doped systems. The average is taken over the 4 different adsorption sites in each system. The lower 4 rows show the hydrogen adsorption on the systems doped with the 4-atom Ti/Co clusters, and the average is taken over the two equivalent cluster centers.

Gas	$E_{ad}^{ave}$ Ti/Co-BC_6_N (eV)
NO_2_	2.8/2.5
NO	3.5/2.6
NH_3_	1.4/1.3
CO_2_	1.3/0.5
N_2_	0.77/0.63
O_2_	4.49/2.78
1H_2_	0.36/0.41
2H_2_	0.31/0.11
1H_2_@4Ti/Co-BC_6_N	1.48/0.40
2H_2_@4Ti/Co-BC_6_N	0.85/1.18
3H_2_@4Ti/Co-BC_6_N	1.06/0.84
4H_2_@4Ti/Co-BC_6_N	0.88/0.70

## Data Availability

The data presented in this study are available in article.

## Supplementary Materials

The following supporting information can be downloaded at: https://www.mdpi.com/article/10.3390/molecules30091873/s1, Figure S1: DOS/projected DOS (PDOS) of: (a) NO@_TB_BC_6_N, (b) NO@_TN_BC_6_N, (c) NO@_H1_BC_6_N and (d) NO@_H2_BC_6_N. Figure S2: DOS/projected DOS (PDOS) of: (a) NO_2_@_TB_BC_6_N, (b) NO_2_@_TN_BC_6_N, (c) NO_2_@_H1_BC_6_N and (d) NO_2_@_H2_BC_6_N. Figure S3: DOS/projected DOS (PDOS) of: (a) CO_2_@_TB_BC_6_N, (b) CO_2_@_TN_BC_6_N, (c) CO_2_@_H1_BC_6_N and (d) CO_2_@_H2_BC_6_N. Figure S4: DOS/projected DOS (PDOS) of: (a) NH_3_@_TB_BC_6_N, (b) NH_3_@_TN_BC_6_N, (c) NH_3_@_H1_BC_6_N and (d) NH_3_@_H2_BC_6_N. Figure S5: The charge density of (a) NO@Ti_B_, (b) NO@Ti_C1_, (c) NO@Ti_C2_ and (d) NO@Ti_N_ respectively from left to right. Isosurface yellow (blue) color represents higher (lower) charge density. Figure S6: The charge density of NO_2_@Ti_B_, NO_2_@Ti_C1_, NO_2_@Ti_C2_ and NO_2_@Ti_N_ respectively from left to right. Isosurface yellow (blue) color represents higher (lower) charge density. Figure S7: The charge density of CO_2_@Ti_B_, CO_2_@Ti_C1_, CO_2_@Ti_C2_ and CO_2_@Ti_N_ respectively from left to right. Isosurface yellow (blue) color represents higher (lower) charge density. Figure S8: The charge density of NH_3_@Ti_B_, NH_3_@Ti_C1_, NH_3_@Ti_C2_ and NH_3_@Ti_N_ respectively from left to right. Isosurface yellow (blue) color represents higher (lower) charge density. Figure S9: The charge density of NO@Co_B_, NO@Co_C1_, NO@Co_C2_ and NO@Co_N_ respectively from left to right. Isosurface yellow (blue) color represents higher (lower) charge density. Figure S10: The charge density of NO_2_@Co_B_, NO_2_@Co_C1_, NO_2_@Co_C2_ and NO_2_@Co_N_ respectively from left to right. Isosurface yellow (blue) color represents higher (lower) charge density. Figure S11: The charge density of CO_2_@Co_B_, CO_2_@Co_C1_, CO_2_@Co_C2_ and CO_2_@Co_N_ respectively from left to right. Isosurface yellow (blue) color represents higher (lower) charge density. Figure S12: The charge density of NH_3_@Co_B_, NH_3_@Co_C1_, NH_3_@Co_C2_ and NH_3_@Co_N_ respectively from left to right. Isosurface yellow (blue) color represents higher (lower) charge density.

## Abbreviations

The following abbreviations are used in this manuscript:

MDPI	Multidisciplinary Digital Publishing Institute
DOAJ	Directory of open access journals
TLA	Three letter acronym
LD	Linear dichroism
