# Supplementary material for: Investigating the Electronic and Molecular Adsorption Properties of Ti/Co-Doped Boron Carbon Nitride
Source: Molecules. 2025 Apr 22;30(9):1873. doi: 10.3390/molecules30091873 (PMC12073601; doi:10.3390/molecules30091873)
Supplement: Supplementary file 1 [file molecules-30-01873-s001.zip › molecules-3367196-supplementary.pdf]

Supplementary Information (SI)
Investigating the Electronic and Molecular Adsorption Properties of Ti/Co-doped Boron Carbon Nitride

Nada M. Alghamdi[1], Hind M. Al-qahtani[2], Amal Alkhaldi[1],
Mohamed M. Fadlallah[3,*] and Ahmed A. Maarouf[4†]

[1] *Department of Physics, College of Science,*
Imam Abdulrahman Bin Faisal University, Dammam 31441, Saudi Arabia

[2] *Department of Physics, College of Science and Humanities,*
Imam Abdulrahman Bin Faisal University, Jubail 35811, Saudi Arabia

[3] *Physics Department, Faculty of Science,*
Benha University, Benha 13518, Egypt and

[4] *Department of Physics, Faculty of Basic Sciences,*
German University in Cairo, New Cairo City 11835, Egypt

[*] mohamed.fadlallah@fsc.bu.edu.eg

[†] ahmed.maarouf@guc.edu.eg

I. DOS/PDOS ON PRISTINE BC_6N

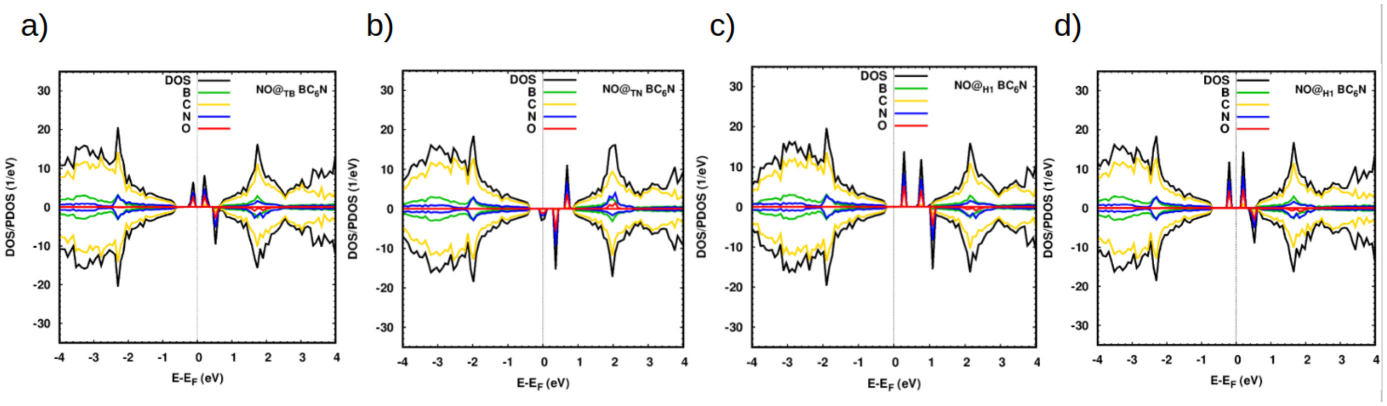

Figure S1. DOS/projected DOS (PDOS) of: (a) $NO@_{TB}BC_6N$, (b) $NO@_{TN}BC_6N$, (c) $NO@_{H1}BC_6N$ and (d) $NO@_{H2}BC_6N$.

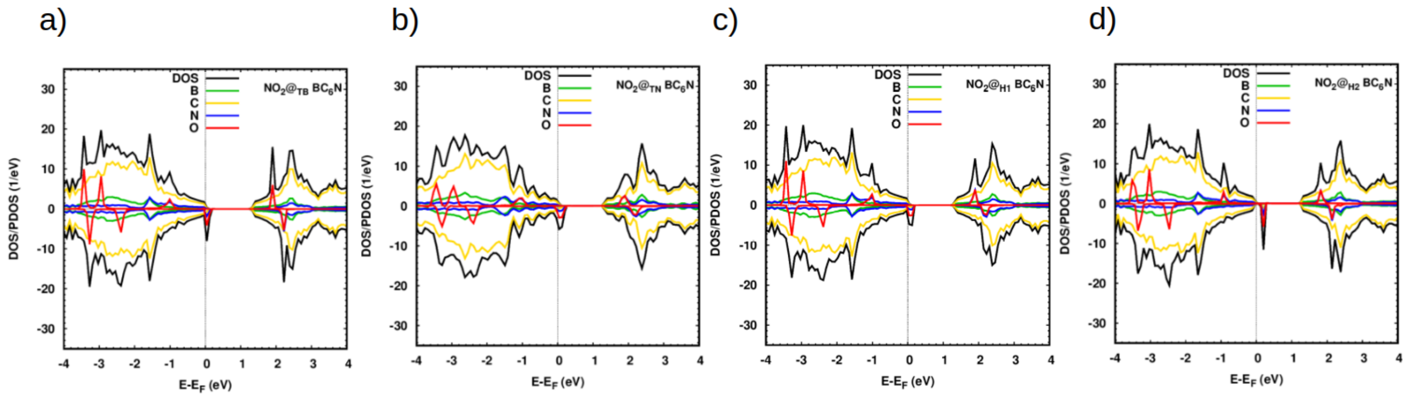

Figure S2. DOS/projected DOS (PDOS) of: (a) $NO_2@_{TB}BC_6N$, (b) $NO_2@_{TN}BC_6N$, (c) $NO_2@_{H1}BC_6N$ and (d) $NO_2@_{H2}BC_6N$.

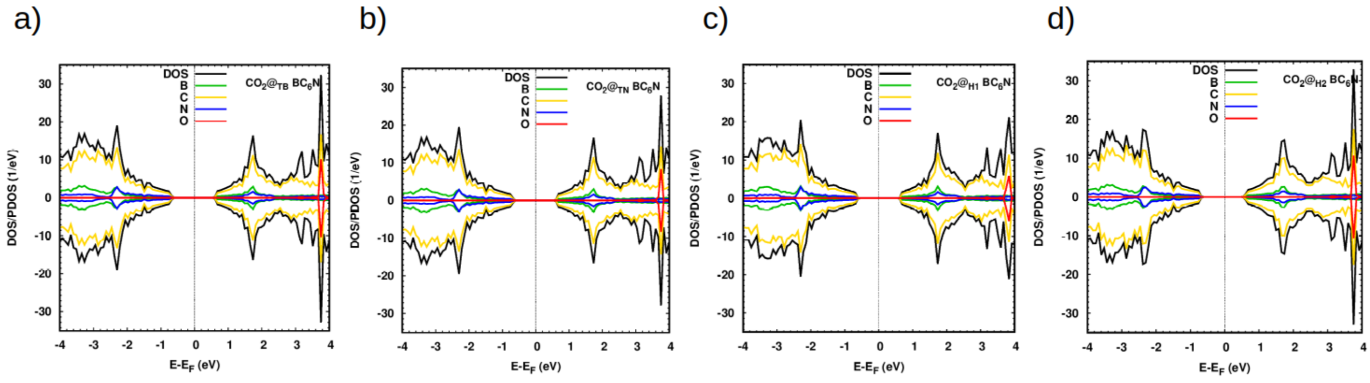

Figure S3. DOS/projected DOS (PDOS) of: (a) $CO_2@_{TB}BC_6N$, (b) $CO_2@_{TN}BC_6N$, (c) $CO_2@_{H1}BC_6N$ and (d) $CO_2@_{H2}BC_6N$.

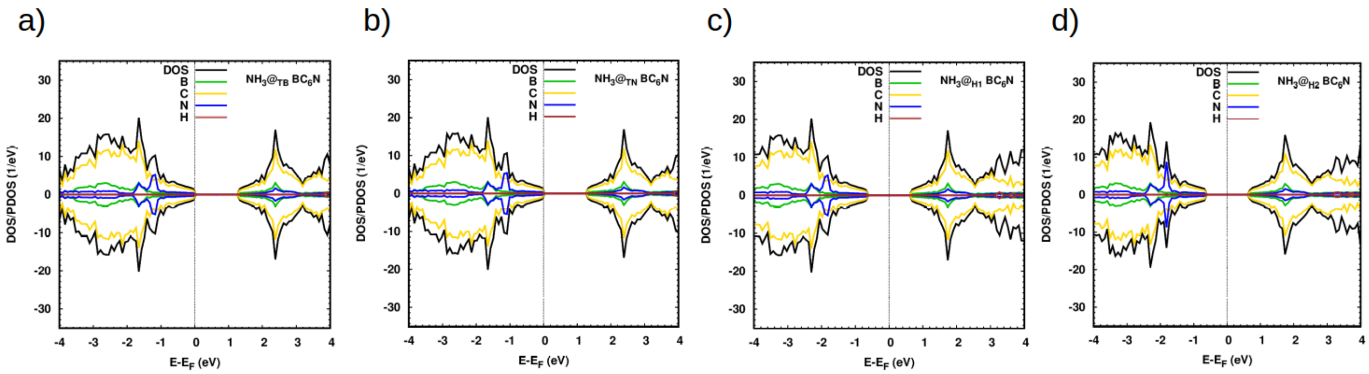

Figure S4. DOS/projected DOS (PDOS) of: (a) $NH_3@_{TB}BC_6N$, (b) $NH_3@_{TN}BC_6N$, (c) $NH_3@_{H1}BC_6N$ and (d) $NH_3@_{H2}BC_6N$.

II. CHARGE DENSITY DIFFERENCE MAPS OF MOLECULES ON DOPED SYSTEMS

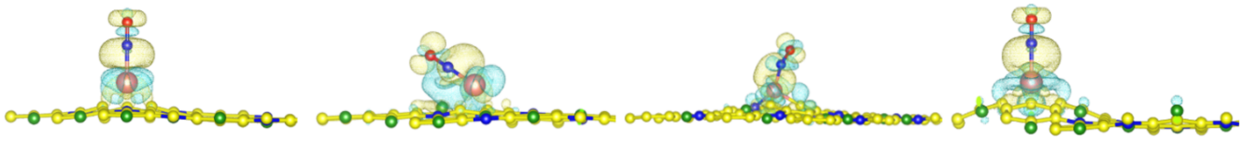

Figure S5. The charge density of NO@Ti$_B$, NO@Ti$_{C1}$, NO@Ti$_{C2}$ and NO@Ti$_N$. Isosurface yellow (blue) color represents higher (lower) charge density.

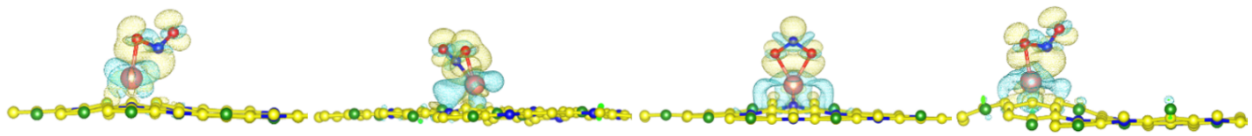

Figure S6. The charge density of NO_2@Ti_B, NO_2@Ti_{C1}, NO_2@Ti_{C2} and NO_2@Ti_N respectively from left to right. Isosurface yellow (blue) color represents higher (lower) charge density.

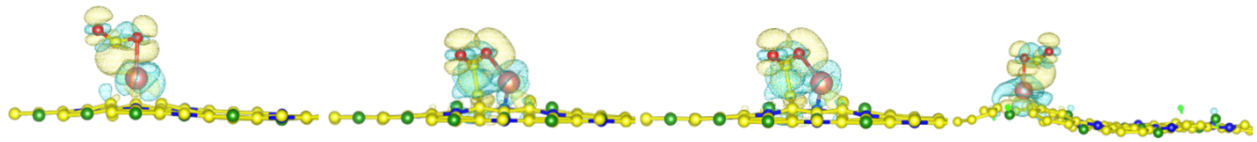

Figure S7. The charge density of CO_2@Ti_B, CO_2@Ti_{C1}, CO_2@Ti_{C2} and CO_2@Ti_N respectively from left to right. Isosurface yellow (blue) color represents higher (lower) charge density.

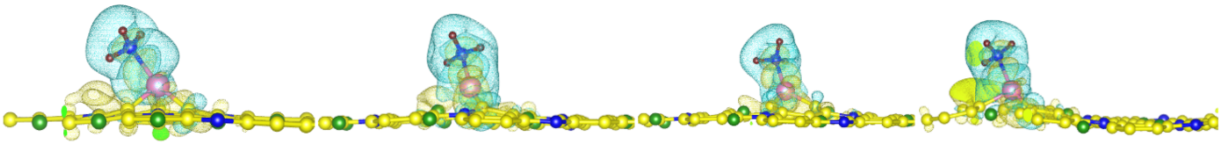

Figure S8. The charge density of NH_3@Ti_B, NH_3@Ti_{C1}, NH_3@Ti_{C2} and NH_3@Ti_N respectively from left to right. Isosurface yellow (blue) color represents higher (lower) charge density.

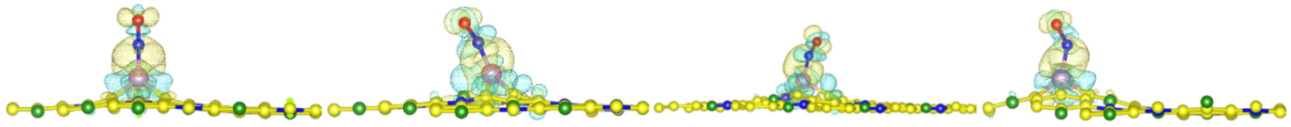

Figure S9. The charge density of NO@Co_B, NO@Co_{C1}, NO@Co_{C2} and NO@Co_N respectively from left to right. Isosurface yellow (blue) color represents higher (lower) charge density.

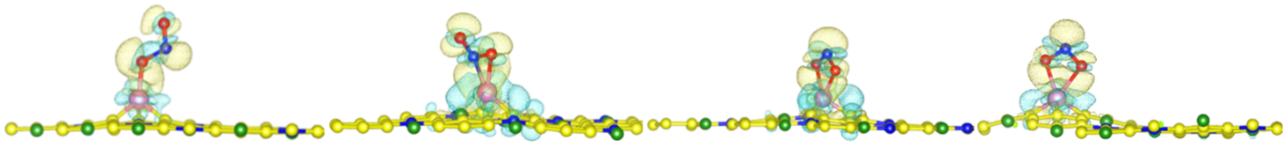

Figure S10. The charge density of NO_2@Co_B, NO_2@Co_{C1}, NO_2@Co_{C2} and NO_2@Co_N respectively from left to right. Isosurface yellow (blue) color represents higher (lower) charge density.

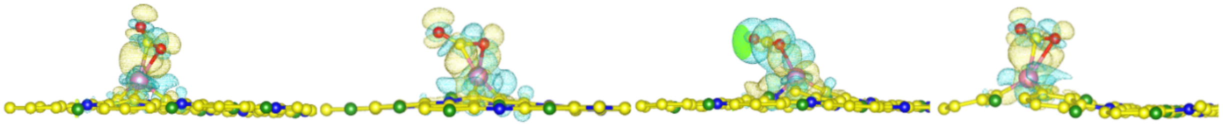

Figure S11. The charge density of CO_2@Co_B, CO_2@Co_{C1}, CO_2@Co_{C2} and CO_2@Co_N respectively from left to right. Isosurface yellow (blue) color represents higher (lower) charge density.

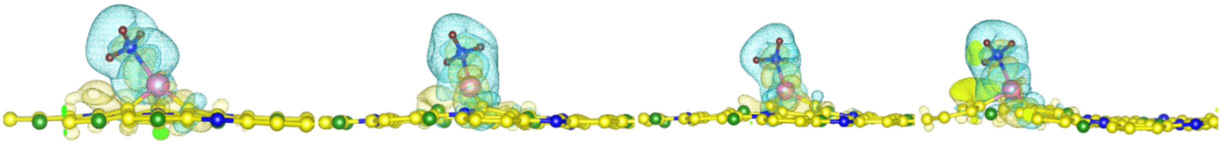

Figure S12. The charge density of NH_3@Co_B, NH_3@Co_{C1}, NH_3@Co_{C2} and NH_3@Co_N respectively from left to right. Isosurface yellow (blue) color represents higher (lower) charge density.
